# Individual and combined effects of dietary vitamin intake on cognitive function in elderly adults: the potential mediating role of serum neurofilament light chain levels

**DOI:** 10.3389/fnut.2025.1485648

**Published:** 2025-01-29

**Authors:** Zhikui Zhou, Baiyun Fan, Qiang Chen, Xuezhong Li, Xianjin Ke

**Affiliations:** ^1^Department of Neurology, Affiliated People's Hospital of Jiangsu University, Zhenjiang, China; ^2^Department of Neurology, Affiliated Hospital of Jiangsu University, Zhenjiang, China

**Keywords:** cognitive function, Bayesian kernel machine regression, vitamins, vitamin K, neurofilament light chain

## Abstract

**Background:**

Vitamins are essential micronutrients for the prevention and treatment of neurodegenerative diseases. The objectives of the present study were to evaluate the association between dietary vitamin intake and cognitive function in elderly adults and to explore the potential impact of serum neurofilament light chain (NfL) concentration.

**Methods:**

Data from 468 elderly individuals, including information on the dietary consumption of 10 vitamins, were used. Cognitive performance was assessed according to a composite Z-score of the Animal Fluency Test (AFT), Consortium to Establish a Registry for Alzheimer’s Disease (CERAD), and Digit Symbol Substitution Test (DSST). Serum NfL levels were measured using a highly sensitive immunoassay. Bayesian kernel machine regression (BKMR) models were used to estimate the combined effects of vitamin mixtures on cognitive function.

**Results:**

In both single- and multiple-vitamin models, individuals with a higher intake of dietary vitamin K exhibited greater global cognitive function, compared to those with a lower vitamin intake. BKMR revealed positive associations between vitamin mixtures and global cognitive function, AFT Z-scores, and DSST Z-scores. Individuals in the third vitamin K intake tertile exhibited lower serum NfL levels than those in the first tertile (regression coefficient, β = −0.16 [95% confidence interval −0.29 to −0.02]; *p* = 0.023). Serum NfL levels mediated the association between higher vitamin K intake and global cognitive function (8.73%).

**Conclusion:**

Vitamin mixtures were positively associated with global cognitive function in elderly participants. The association between vitamin K intake and cognitive function may be mediated by serum NfL concentration.

## 1 Introduction

Cognitive function is a crucial factor for maintaining quality of life and can be affected by aging and neurodegenerative disease(s). One of the most common diseases attributed to age-related decline in cognitive function is dementia (1). Dementia is a primary cause of death and disability among elderly adults worldwide (2). It has been estimated that the global number of cases of dementia was 57.4 million in 2019 and is projected to be 152.8 million in 2050 (3). Generally, dementia evolves from cognitive decline or impairment, and the transition to the disease is irreversible (4). Cognitive decline can be triggered by various pathological, behavioral, and environmental factors (3). As such, identifying modifiable risk factors and developing interventional strategies to prevent or, at least mitigate, cognitive decline in elderly individuals have significant public health implications.

Diet can act as a modifiable risk factor and nutrients may have an impact(s) on the decline of cognitive function (5). Vitamins are essential micronutrients for normal nervous system functioning and neurological wellbeing. By regulating DNA synthesis and methylation, and maintaining phospholipids, vitamins exert pleiotropic effects on neuronal structure, function, and neuroendocrine pathways (6). The neurofilament light chain (NfL), a component of the axonal cytoskeleton, has been identified as a marker of neuro-axonal damage in various neurological disorders, including Alzheimer’s disease (7, 8). The correlation between NfL in the cerebrospinal fluid and blood suggests that blood-based NfL can serve as a biomarker for monitoring neurological disorders (9).

Epidemiological studies have demonstrated that the antioxidant and neuroprotective properties of vitamins may contribute to neural integrity and cognitive preservation (10, 11). Several studies have generated consistent evidence supporting folate (vitamin B_9_) (12), and putative evidence for other vitamins associated with cognitive impairment in the general population (13, 14). However, the effects of vitamin combinations on cognitive function remain unclear. As such, we hypothesized that serum NfL levels may be involved in the association between individual vitamin intake and cognitive function. The aims of the present study were, therefore, to examine the associations between the intake of multiple vitamins and cognitive function among elderly participants, and to evaluate the potential impact of NfL on these associations.

## 2 Methods

### 2.1 Study design and population

The current analysis sourced publicly available data from the National Health and Nutrition Examination Survey (NHANES) database. Among the critical objectives of the NHANES was to evaluate the nutritional status and health of a large, representative sample of the United States population. Given the availability of data, the NHANES 2013–2014 cycle was used (15). Participants provided written informed consent before enrollment, and as such, additional consent requirements were waived. The study protocol (No. #2011-17) was reviewed and approved by the NCHS Research Ethics Review Board.

A total of 10,175 participants were enrolled in the NHANES 2013–2014 cycle, and data from elderly participants ≥60 years of age (*n* = 1,841) were extracted and included in the present study. Participants who lacked cognitive assessment data were excluded (*n* = 268), as were those without dietary information (*n* = 133), and/or serum NfL measurement data (*n* = 972) (Supplementary Figure S1).

### 2.2 Calculation of vitamin intake(s)

For NHANES participants, two 24-h dietary recall interviews were conducted (16): the first interview was in-person at the mobile examination center, and the second was via telephone after 3–10 days. The vitamin intake of each individual was calculated based on two 24-h dietary intakes and dietary supplements according to the United States Department of Agriculture Food and Nutrient Database (17). This database contains extensive information used for coding individual foods and beverages, as well as the portion sizes reported by participants. It also provides the nutrient values necessary for calculating nutrient intake(s). Dietary vitamin intake was assessed by averaging the data from two 24-h dietary and supplementation recalls. A panel of 10 vitamins was incorporated into the present analysis.

### 2.3 Serum NfL concentration

Serum samples were collected and frozen for further analysis. A high-sensitivity immunoassay was used to quantify serum NfL concentrations (15). Briefly, the samples were incubated with antibodies labeled with acridinium ester (AE), which specifically binds to the NfL antigen. Paramagnetic particles (PMPs) coated with a capture antibody were added to the sample, thus forming complexes formed by the antigen bound to both the AE-labeled antibodies and PMPs. Any unbound AE-labeled antibodies were separated and eliminated. Finally, acids and bases were added to generate chemiluminescence, enabling light emission measurements. As reported on the NHANES website, the analysis followed strict quality control and assurance procedures. The limit of quantification for NfL was 3.9 pg./mL.

### 2.4 Cognitive function

During the survey, the Consortium to Establish a Registry for Alzheimer’s Disease Word Learning test (CERAD W-L), the Animal Fluency test (AFT), and the Digit Symbol Substitution test (DSST) were administered to participants ≥60 years of age. As previously reported (18, 19), a combination of these tests has been widely used to assess individual cognitive performance, including verbal, memory, and executive abilities. The CERAD W-L, AFT, and DSST scores were standardized using the Z-score method (20), and a composite sum Z-score was used to assess individual global cognitive function (21, 22).

### 2.5 Covariates

Known or suspected influential factors of vitamin consumption and cognitive function were screened based on the previous literature (23, 24) and the directed acyclic graph method (Supplementary Figure S2). The final models included sex, age, ethnicity, education, family income-to-poverty ratio (PIR), body mass index (BMI), smoking status, alcohol consumption, physical activity levels, diabetes, hypertension, and energy intake. Smoking status was assessed using a cutoff of 100 cigarettes in life as never smoked, former smoker, or current smoker, while 12 alcoholic beverages/year were used as the cutoff to define alcohol consumption. Physical activity was measured according to moderate/vigorous recreational activity for ≥10 min continuously per week (25). Diabetes was evaluated by self-reported diabetes history and relevant medicine taken in combination with laboratory investigations, including a glycated hemoglobin (HbA1c) level > 6.5%, fasting blood glucose level ≥ 7.0 mmol/L, and a 2 h plasma glucose level ≥ 11.1 mmol/L (26). Similarly, hypertension was assessed according to measurements of systolic blood pressure (SBP) and diastolic blood pressure (DBP), self-reported history of hypertension, and the use of antihypertensive medication (27).

### 2.6 Statistical analysis

Participant characteristics are expressed as mean (standard deviation [SD]) and frequency (proportion). Vitamin intake was transformed into categorical variables (tertiles) due to non-linear dose–response relationships, as described previously (28). Serum NfL concentrations were natural logarithm (ln)-transformed due to skewed distribution. Multiple imputations with chained equations were applied to the small number of missing covariates.

Multivariable linear regression models were used to explore the associations between vitamin intake, sum Z-score, and serum NfL concentrations. The variance inflation factor (VIF) was used to assess collinearity in the linear regression models with no multicollinearity. Multivariable generalized additive models (GAMs) were used to estimate potential non-linear associations between vitamin consumption and cognitive function. The Wald test was used to test the significance of the models. Bayesian kernel machine regression (BKMR) models were used to assess the combined effects of the vitamin mixtures on cognitive function (29). BKMR is a novel approach for assessing dose–response relationships, joint effects of vitamin mixtures, and individual effects among vitamin mixtures (29). Mediation analysis was performed to assess the potential mediating role of serum NfL levels in the relationship between vitamin intake and cognitive function (22).

In the sensitivity analysis, several factors were considered to confirm the robustness of the results, including potential confounders such as beta-carotene, the relationship between serum NfL and global cognitive function, and single vitamin intake in the source population.

Statistical analyses were performed using R version 4.3.2 (R Foundation for Statistical Computing, Vienna, Austria). Differences with a *p*-value of < 0.05 were considered to be statistically significant.

## 3 Results

### 3.1 Participant characteristics

The general characteristics of the 468 elderly individuals (219 male [46.8%]) included in this study are summarized in Table 1. The mean (± SD) age and BMI were 66.3 ± 4.4 years and 29.6 ± 6.8 kg/m^2^, respectively. Among the participants, 228 (48.7%) were non-Hispanic white, and 249 (53.2%) had a college degree or above. The proportions of participants with diabetes and hypertension were 34.4 and 76.3%, respectively.

**Table 1:** Descriptive statistics of general characteristics of 468 participants from NHANES 2013–2014.

Characteristics	*N* (%)/Mean (SD) *n* = 468	*N* (%)/Mean (SD) *n* = 1,841	*p*-value
**Sex**			
Male	219 (46.8)	874 (47.5)	0.833
Female	249 (53.2)	967 (52.5)	
**Ethnicity**			
Mexican American	55 (11.7)	210 (11.4)	0.608
Other Hispanic	49 (10.5)	154 (8.4)	
Non-Hispanic white	228 (48.7)	896 (48.7)	
Non-Hispanic Black	92 (19.7)	388 (21.1)	
Other ethnicities- including multi-racial	44 (9.4)	193 (10.5)	
**Education**			
Less than high school	114 (24.4)	491 (26.7)	0.324
High school	105 (22.4)	440 (23.9)	
College and above	249 (53.2)	907 (49.3)	
NA’s		3 (0.2)	
**Smoking status**			
Never smoking	231 (49.4)	917 (49.8)	0.686
Former smoking	171 (36.5)	690 (37.5)	
Current smoking	66 (14.1)	232 (12.6)	
NA’s		2 (0.1)	
**Alcohol consumption**			
Yes	139 (29.7)	566 (30.7)	0.100
No	329 (70.3)	1,104 (60.0)	
NA’s		171 (9.3)	
**Physical activity**			
Vigorous or moderate recreational activities	209 (44.7)	756 (41.1)	0.175
No	259 (55.3)	1,085 (58.9)	
**Diabetes**			
Yes	161 (34.4)	437 (23.7)	<0.001
No	307 (65.6)	1,403 (76.2)	
NA’s		1 (0.1)	
**Hypertension**			
Yes	357 (76.3)	1,170 (63.6)	<0.001
No	111 (23.7)	667 (36.2)	
NA’s		4 (0.2)	
Age (year)	66.3 (4.4)	70.0 (6.9)	<0.001
BMI (kg/m^2^)	29.6 (6.8)	29.0 (6.3)	0.137
PIR	2.6 (1.6)	2.5 (1.6)	0.339
Energy intake (kcal)	1,849 (755)	1,836 (835)	0.367
Neurofilament light levels	25.59 (28.33)	25.60 (27.53)	0.997
CERAD W-L	27.03 (6.19)	25.37 (6.85)	<0.001
AFT	17.25 (5.72)	16.43 (5.55)	0.004
DSST	47.97 (17.61)	45.95 (17.23)	0.025
Vitamin A	575.4 (544.0)	529.3 (516.9)	0.089
Vitamin B1	1.33 (0.76)	1.20 (0.78)	<0.001
Vitamin B2	1.75 (1.01)	1.56 (1.04)	<0.001
Vitamin B6	1.70 (1.05)	1.55 (1.15)	0.008
Vitamin B9	423.0 (284.7)	387.0 (292.0)	0.013
Vitamin B12	4.79 (5.30)	4.31 (4.80)	0.067
Vitamin C	72.1 (72.9)	69.2 (71.6)	0.428
Vitamin D	4.44 (5.10)	3.95 (4.53)	0.054
Vitamin E	7.93 (6.64)	7.24 (7.21)	0.042
Vitamin K	107.1 (122.3)	93.5 (116.8)	0.026

N, frequency; %, proportion; SD, standard deviation; NA, not available; BMI, body mass index; PIR, poverty–income ratio; CERAD W-L, Consortium to Establish a Registry for Alzheimer’s Disease Word Learning test; AFT, Animal Fluency test; DSST, Digit Symbol Substitution test.

**Figure 1:**
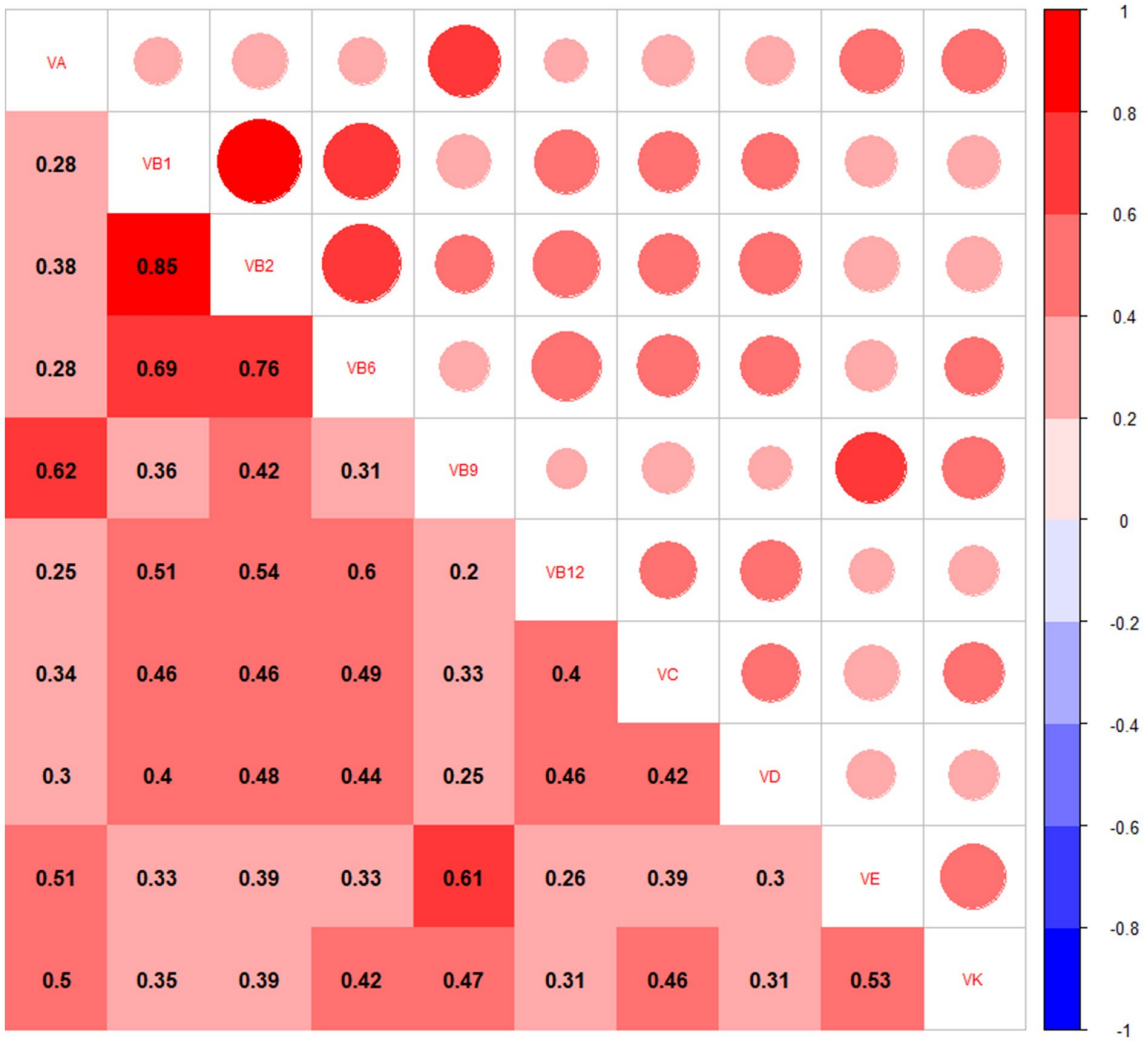
Correlations of dietary vitamin intakes in 468 participants.

The mean CERAD, AFT, and DSST scores were 27.4 ± 6.0, 17.4 ± 5.7, and 48.7 ± 17.4, respectively. The correlations of vitamin intake ranged from low to high based on Pearson’s correlation analyses (Figure 1).

### 3.2 Single vitamin intake and cognitive function

After adjusting for covariates (Table 2), global cognitive function in the third tertile (regression coefficient, β = 0.49 [95% confidence interval (CI) 0.02–0.96]; *p* = 0.041) and the second tertile (β = 0.78 [95% CI 0.33–1.23]; *p* = 0.001) of vitamin A intake were higher than in the first tertile. Global cognitive function in the second vitamin B_1_ intake tertile was significantly higher compared to that in the first tertile (β = 0.58 [95% CI 0.12–1.04]; *p* = 0.013). Higher vitamin B_2_ intake predicted increased global cognitive function (β = 0.58 [95% CI 0.09–1.06]; *p* = 0.020). Global cognitive function was higher in the third vitamin B_9_ intake tertile than that of the first tertile (β = 0.68 [95% CI 0.14–1.21]; *p* = 0.013). Compared to individuals in the first vitamin K intake tertile, those in the third tertile exhibited higher global cognitive function (β = 0.90 [95% CI 0.42–1.39]; *p* < 0.001).

**Table 2:** Associations between global cognitive function and single vitamin intake.

	Crude model					Adjusted model				
	Tertile 1	Tertile 2		Tertile 3		Tertile 1	Tertile 2		Tertile 3	
		β (95%CI)	*p*-value	β (95%CI)	*p*-value		β (95%CI)	*p*-value	β (95%CI)	*p*-value
Vitamin A	Ref.	1.31 (0.79, 1.84)	<0.001	0.93 (0.41, 1.45)	<0.001	Ref.	0.78 (0.33, 1.23)	0.001	0.49 (0.02, 0.96)	0.041
Vitamin B1	Ref.	0.63 (0.10, 1.16)	0.019	0.82 (0.29, 1.34)	0.002	Ref.	0.58 (0.12, 1.04)	0.013	0.36 (−0.12, 0.86)	0.144
Vitamin B2	Ref.	0.74 (0.21, 1.26)	0.006	1.21 (0.69, 1.73)	<0.001	Ref.	0.38 (−0.07, 0.83)	0.101	0.58 (0.09, 1.06)	0.020
Vitamin B6	Ref.	0.50 (−0.02, 1.02)	0.061	1.02 (0.49, 1.54)	<0.001	Ref.	0.28 (−0.17, 0.73)	0.224	0.36 (−0.10, 0.82)	0.127
Vitamin B9	Ref.	1.12 (0.60, 1.63)	<0.001	1.15 (0.63, 1.67)	<0.001	Ref.	0.44 (−0.02, 0.89)	0.063	0.68 (0.14, 1.21)	0.013
Vitamin B12	Ref.	0.61 (0.09, 1.14)	0.021	1.02 (0.50, 1.55)	<0.001	Ref.	0.31 (−0.13, 0.75)	0.162	0.29 (−0.16, 0.73)	0.209
Vitamin C	Ref.	0.47 (−0.05, 0.99)	0.077	1.23 (0.72, 1.76)	<0.001	Ref.	0.20 (−0.22, 0.63)	0.348	0.40 (−0.05, 0.86)	0.081
Vitamin D	Ref.	0.46 (−0.06, 0.99)	0.083	1.04 (0.51, 1.56)	<0.001	Ref.	0.11 (−0.32, 0.54)	0.611	0.14 (−0.31, 0.59)	0.538
Vitamin E	Ref.	0.64 (0.13, 1.15)	0.014	1.65 (1.14, 2.16)	<0.001	Ref.	0.15 (−0.31, 0.61)	0.523	0.45 (−0.10, 1.00)	0.109
Vitamin K	Ref.	0.93 (0.43, 1.44)	<0.001	1.81 (1.30, 2.32)	<0.001	Ref.	0.42 (−0.02, 0.87)	0.063	0.90 (0.42, 1.39)	<0.001

Models were adjusted by age, sex, ethnicity, education, family income-to-poverty ratio, body mass index, marital status, smoking status, alcohol consumption, physical activity, hypertension, diabetes, and energy intake.

Individuals with higher vitamin A and K intakes exhibited higher CERAD Z-scores (Supplementary Table S1). Higher vitamin A, B_1_, B_2_, B_9_, C, and K intake predicted higher AFT Z-scores. The DSST Z-score was higher in participants with a higher intake of vitamins A, B_1_, B_2_, B_9_, and K than in those with a lower intake.

### 3.3 Mixture of vitamin intake and impact on cognitive function

**Figure 2:**
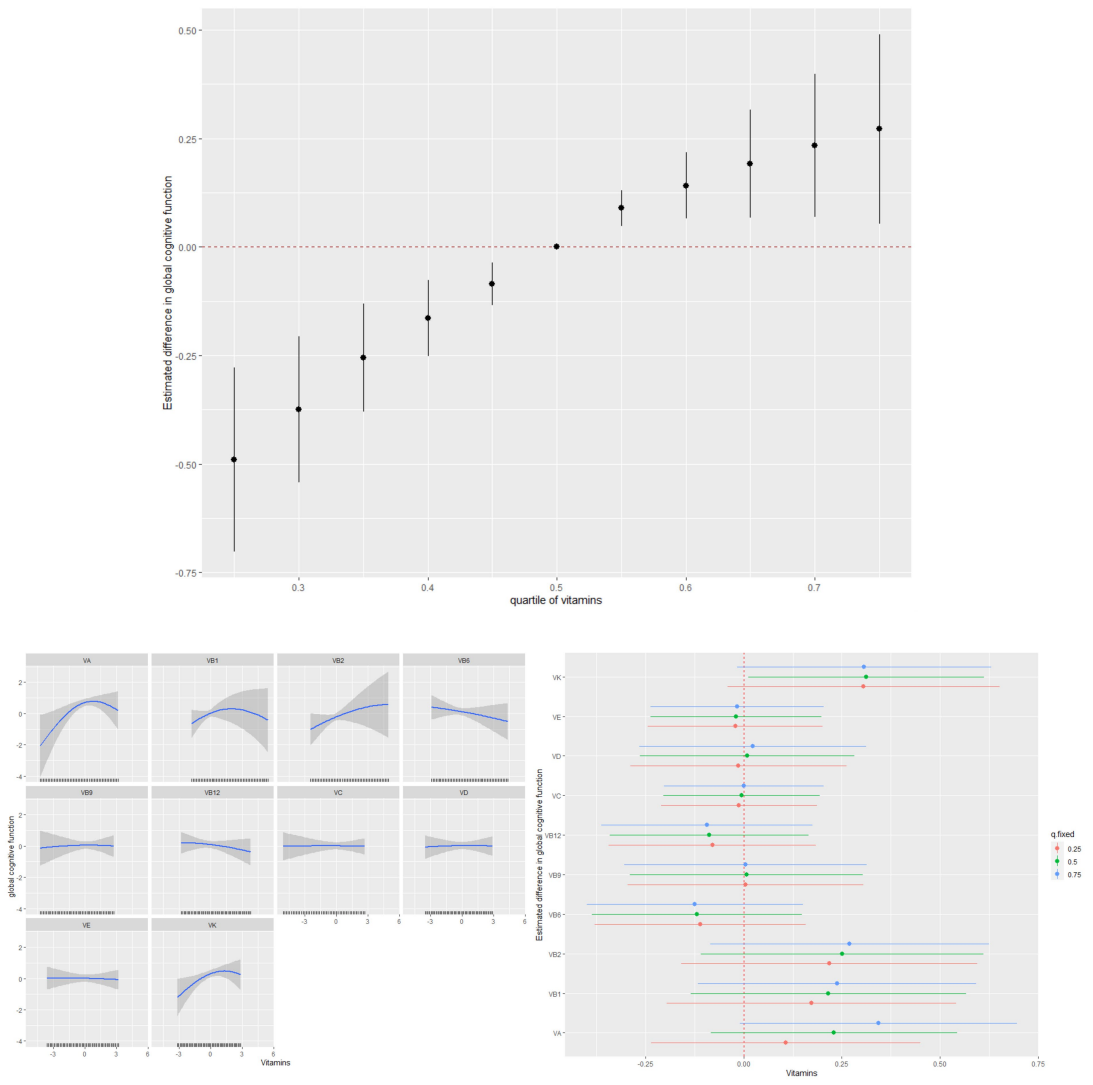
Individual and combined effects of vitamins on cognitive function and dose–response relationships in Bayesian kernel machine regression models.

As shown in Figure 2, the associations of vitamins A, B_1_, B_12_, and K intake and global cognitive function appeared to be non-linear. These non-linear associations were further verified using GAMs (*p* < 0.05) (Supplementary Figure S3). Notably, vitamin intake was positively associated with global cognitive function in the BKMR model. When other vitamins were fixed at the 50th percentile, vitamins A and K levels were marginally and significantly associated with an increase in global cognitive function.

**Figure 3:**
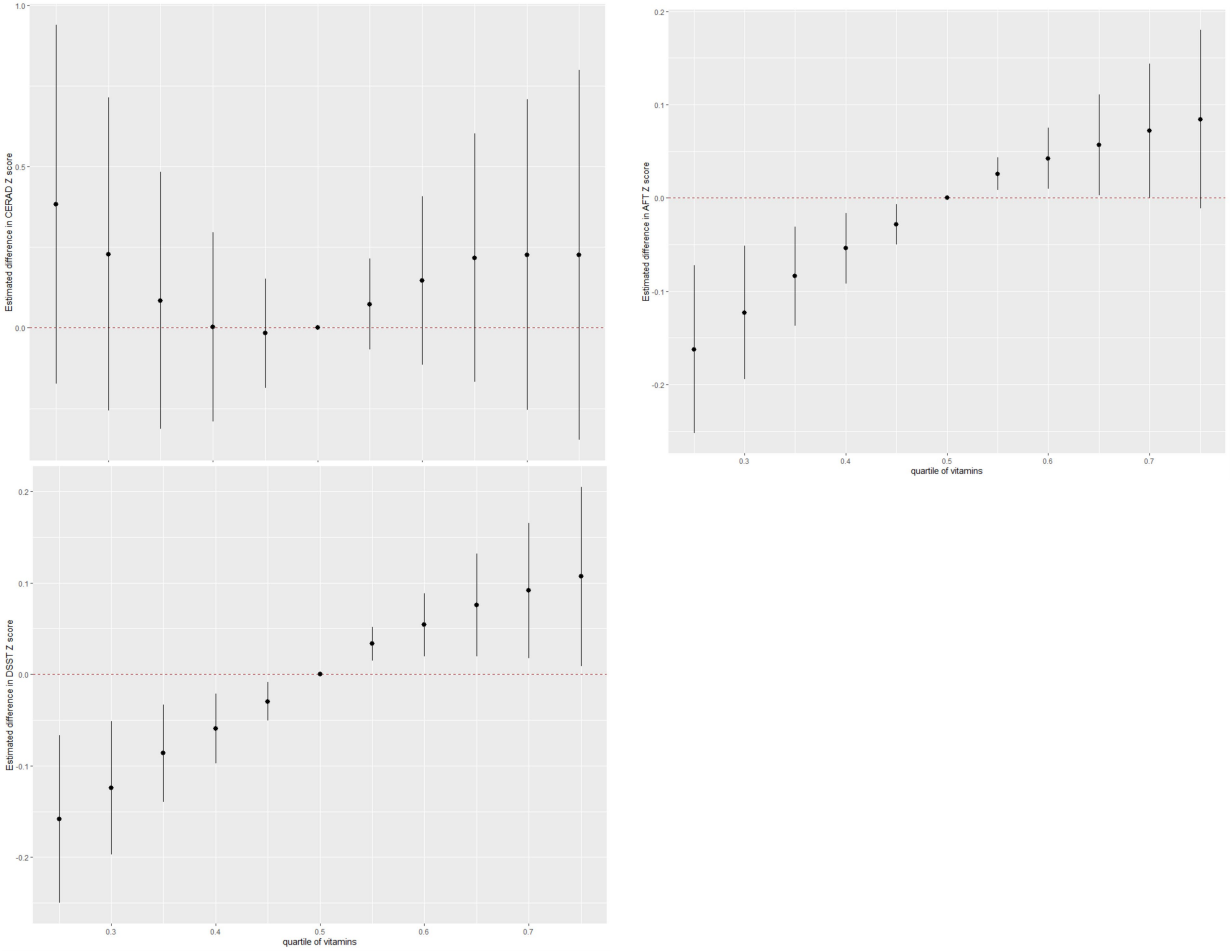
Combined effects of vitamin mixtures on the sub-score of cognitive function.

A combination of vitamins was associated with an increase in AFT and DSST Z-scores but not with the CERAD Z-score (Figure 3).

### 3.4 Individual vitamin intake and serum NfL levels

After adjusting for potential confounders (Table 3), individuals in the third vitamin K intake tertile exhibited lower serum concentrations of NfL than those in the first tertile (β = −0.16 [95% CI −0.29 to −0.02]; *p* = 0.023).

**Table 3:** Associations between serum neurofilament light levels and single vitamin intake.

	Crude model					Adjusted model				
	Tertile 1	Tertile 2		Tertile 3		Tertile 1	Tertile 2		Tertile 3	
		β (95%CI)	*p*-value	β (95%CI)	*p*-value		β (95%CI)	*p*-value	β (95%CI)	*p*-value
Vitamin A	Ref.	−0.01 (−0.13, 0.11)	0.825	0.02 (−0.09, 0.14)	0.722	Ref.	0.02 (−0.10, 0.15)	0.727	0.05 (−0.08, 0.19)	0.727
Vitamin B1	Ref.	−0.09 (−0.21, 0.03)	0.144	−0.12 (−0.24, 0.00)	0.047	Ref.	−0.07 (−0.20, 0.05)	0.264	−0.13 (−0.26, 0.00)	0.057
Vitamin B2	Ref.	−0.02 (−0.14, 0.10)	0.752	−0.10 (−0.22, 0.02)	0.106	Ref.	−0.01 (−0.13, 0.12)	0.926	−0.12 (−0.25, 0.02)	0.096
Vitamin B6	Ref.	−0.04 (−0.17, 0.07)	0.425	−0.11 (−0.23, 0.01)	0.079	Ref.	−0.05 (−0.18, 0.07)	0.382	−0.12 (−0.25, 0.01)	0.076
Vitamin B9	Ref.	−0.08 (−0.21, 0.03)	0.158	−0.06 (−0.18, 0.05)	0.288	Ref.	−0.03 (−0.16, 0.09)	0.629	0.01 (−0.14, 0.16)	0.872
Vitamin B12	Ref.	−0.02 (−0.15, 0.09)	0.651	−0.09 (−0.21, 0.03)	0.144	Ref.	−0.02 (−0.14, 0.10)	0.747	−0.08 (−0.21, 0.04)	0.166
Vitamin C	Ref.	0.03 (−0.08, 0.16)	0.564	−0.07 (−0.19, 0.05)	0.256	Ref.	0.06 (−0.05, 0.18)	0.289	−0.04 (−0.16, 0.09)	0.563
Vitamin D	Ref.	0.02 (−0.11, 0.14)	0.808	−0.04 (−0.16, 0.09)	0.568	Ref.	0.03 (−0.09, 0.15)	0.633	−0.02 (−015, 0.11)	0.761
Vitamin E	Ref.	−0.04 (−0.16, 0.08)	0.469	−0.14 (−0.26, −0.02)	0.022	Ref.	−0.03 (−0.16, 0.09)	0.634	−0.09 (−0.25, 0.06)	0.223
Vitamin K	Ref.	−0.11 (−0.23, 0.01)	0.071	−0.14 (−0.26, −0.02)	0.018	Ref.	−0.11 (−0.23, 0.02)	0.101	−0.16 (−0.29, −0.02)	0.023

Models were adjusted by age, sex, ethnicity, education, family income-to-poverty ratio, body mass index, marital status, smoking status, alcohol consumption, physical activity, hypertension, diabetes, and energy intake.

### 3.5 Mediation analysis

**Figure 4:**
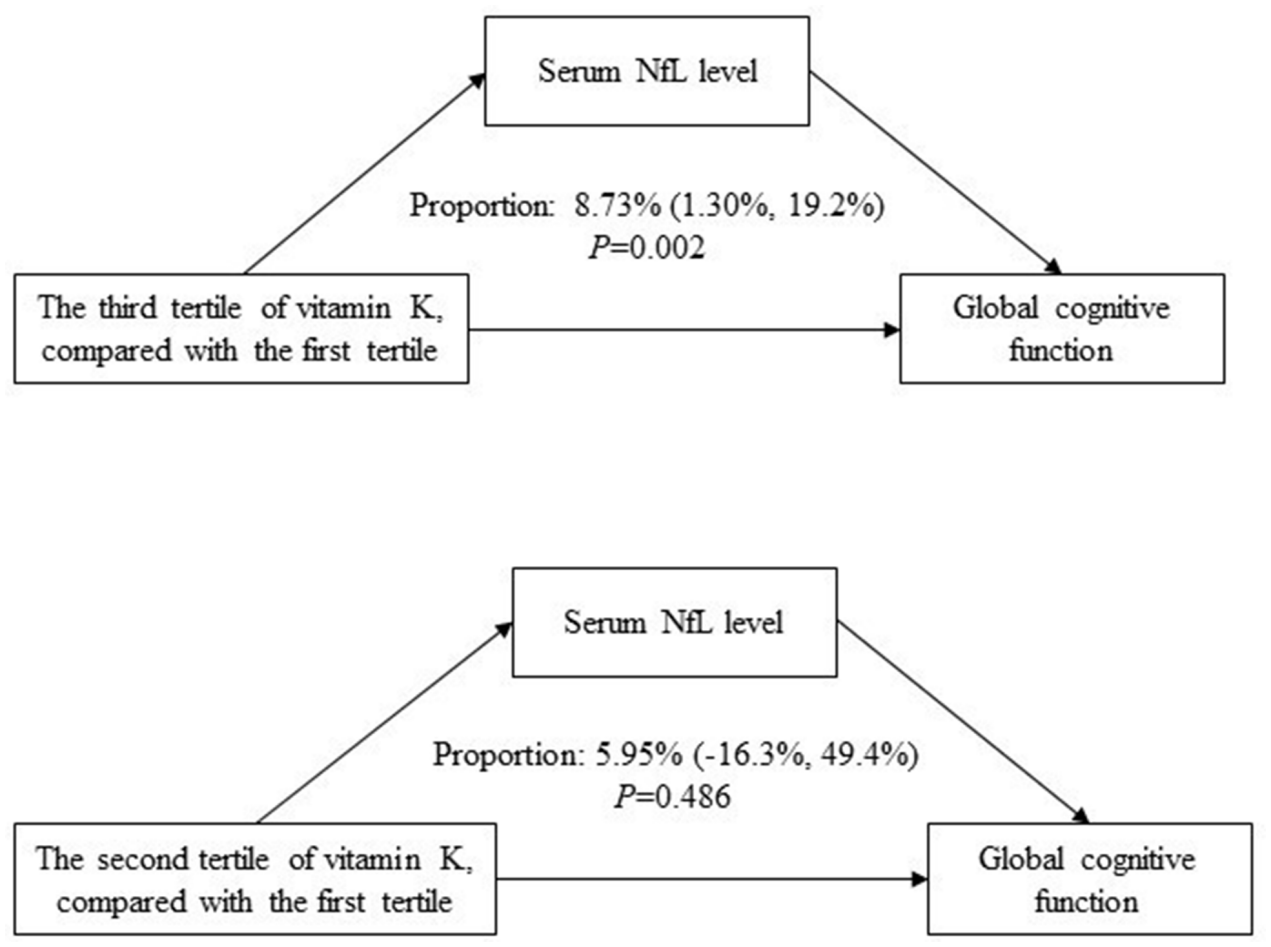
Mediating effects of serum NfL concentration on the association between vitamin K and global cognitive function.

As shown in Figure 4, serum NfL concentration mediated the association between vitamin K intake (the third tertile compared to the first tertile) and global cognitive function, with a mediating effect of 8.73% (95% CI 1.30–19.2%; *p* = 0.002).

### 3.6 Sensitivity analysis

After adjusting for beta-carotene, the main findings were, in large part, consistent with those obtained without adjustment (Supplementary Table S2). The associations between serum NfL levels or global cognitive function and single vitamin intake in the source population were similar to those in the analyzed population (Supplementary Table S3).

## 4 Discussion

In this cross-sectional analysis, we observed that in both single and mixed vitamin models, vitamin A and vitamin K intakes were associated with higher global cognitive function. The most remarkable finding of the current study was that the intake of the 10 vitamin mixtures was associated with an increase in global cognitive function. Higher vitamin K intake predicted a decrease in serum NfL concentration. Serum NfL concentration mediates the relationship between vitamin K intake and global cognitive function. To the best of our knowledge, this is the first study to explore the potential relationship between multiple vitamin intake and cognitive function among elderly individuals in the United States.

Vitamin B plays a crucial role in preventing cognitive decline during various neurological processes (10). Previous studies have demonstrated that the B vitamins can reduce the risk of dementia by regulating homocysteine (Hcy) levels or affecting amino acids linked to neurodegenerative diseases (12). Vitamins B_1_, B_6_, and B_12_ are involved in the synthesis of myelin and neurotransmitters that exert their neuroprotective effects (30). Regarding epidemiological evidence, Wang et al. (12) summarized 95 studies comprising 46,175 participants and showed that vitamin B supplementation may reduce the risk for cognitive decline and even incident dementia. Among these vitamins, the associations between vitamins B_6_, B_9_, and B_12_ and cognitive function have been extensively investigated (12, 24). However, the neurological effects of vitamins B_1_ and B_2_ are relatively limited. The latest studies using the NHANES database reported that dietary vitamin B_1_ and vitamin B_2_ intakes were associated with better cognitive performance among elders in the United States (23, 31). Consistent with previous findings, we observed that higher vitamin B_1_, B_2_, and B_9_ intake was associated with increased cognitive function in single vitamin models.

Antioxidant vitamins, such as vitamins A, C, and E, were included in the calculation of novel indices to assess the relationship between antioxidant indicators and human health. A cross-sectional analysis revealed that the composite dietary antioxidant index, which is calculated from three minerals and three vitamins (A, C, and E), was associated with an elevation in global cognitive function among elders in the United States (21). Similarly, another study found that dietary total antioxidant capacity—including vitamins A, C, and E—was associated with higher IRT, AFT, and DSST scores (32). The oxidative balance score, generated from 16 nutrients and 4 lifestyle components, was positively correlated with global cognitive function in elderly adults (33). Our findings suggest that higher vitamin A intake predicted better cognitive performance in both single- and multiple-vitamin models than in lower vitamin A intake.

Evidence supporting the association between vitamin K intake and cognitive function has recently increased. Sphingolipid metabolism is a crucial pathway through which vitamin K affects age-related brain function (34). Similar to other vitamins, vitamin K has antioxidant and anti-inflammatory effects related to the regulation of cognitive function. In a multicenter interventional study involving 5,533 older participants, higher dietary vitamin K intake was associated with better cognitive function (35). Menaquinone-4, a form of vitamin K in the brain, is associated with a lower risk for dementia and mild cognitive impairment, implying its involvement of vitamin K in the neuropathology of cognitive decline (36). Serum phylloquinone concentration, a marker of vitamin K, is associated with higher cognitive performance in older adults, especially memory (37). Similarly, our findings indicate that vitamin K is associated with higher global cognitive function in elderly individuals in the United States in single- and multiple-vitamin models. Although the exact mechanism by which vitamin K affects cognitive function is not yet clear, several potential mechanisms have been proposed to account for the protective effect of vitamin K on cognitive function, including participation in sphingolipid metabolism (38), anti-inflammation (39), anti-oxidative stress (40), and the activation of vitamin K-dependent proteins such as growth-arrest specific 6 (Gas6) and protein S (41).

Vitamin D can exert neurological effects on cognitive function through various biological mechanisms including oxidative stress, calcium homeostasis, and inflammatory pathways (42). Evidence from interventional and observational studies has shown inconsistent associations between vitamin D and cognitive function (13). In the Framingham Heart Study, no association between serum 25-hydroxyvitamin D concentration and Alzheimer’s disease was observed (43). Similarly, in the current study, we did not observe any association between vitamin D intake and cognitive function among elderly individuals.

To date, studies investigating the association between vitamin intake and serum NfL concentration have been limited. Similar to our findings, a previous study using data from the 2013–2014 cycle of the NHANES cycles also observed that higher vitamin K intake was associated with decreased serum NfL concentrations (44). Several clinical trials involving patients with multiple sclerosis reported a relationship between vitamin D supplementation and serum NfL concentration with conflicting findings (45). Insufficient intake of vitamin K may influence the activation of vitamin K-dependent on vitamin K (such as Gas 6 and protein S) and the synthesis of sphingolipids within the nervous system (46–48). Both Gas 6 and protein S exhibit diverse neuroprotective attributes, including the promotion of cell proliferation, prevention of apoptosis, and reduction of inflammation, which may mitigate neuronal damage and a subsequent decline in NfL levels. In the current study, we found that the serum NfL concentration acted as a mediator between vitamin K intake and global cognitive function. Our findings highlight that vitamin K intake may be involved in neurological protection, although the exact biological mechanisms require further investigation.

The primary strength of our study was the assessment of a panel of 10 vitamins, which may provide a basis for future studies investigating the impact of vitamin combinations in relation to cognitive function. Another advantage of the present study is that we investigated the potential involvement of serum NfL concentration in the association between vitamin intake and cognitive function. However, this study also had several limitations, the first of which was its cross-sectional design, which precluded us from drawing causal inferences. In addition, vitamin intake was obtained through dietary evaluation, which may have caused misclassification due to various starting values, especially for vitamin D. Meanwhile, we cannot exclude the possibility of residual confounding factors, such as the state of the intestine (49), creatine level (50), or air pollution (51), although a series of potential confounders were adjusted for in the statistical models. As such, the neurological effects of these combinations remain unclear. Finally, using a 24-h dietary recall interview to assess vitamin intake in exposure assessments may have been subject to recall bias.

## 5 Conclusion

A higher intake of vitamin K is associated with increased cognitive function among elderly individuals in the United States. A mixture of 10 vitamins was associated with an increase in global cognitive function. The association between vitamin K intake and cognitive function may be mediated by serum NfL concentration. These findings emphasize the importance of balanced vitamin intake in maintaining cognitive wellbeing in an aging population. These results highlight the need to develop interventional strategies to prevent cognitive decline in the geriatric population.

## Data Availability

The datasets presented in this study can be found in online repositories. The names of the repository/repositories and accession number(s) can be found below: https://www.cdc.gov/nchs/nhanes/index.htm.
