# Supplementary material for: Individual and combined effects of dietary vitamin intake on cognitive function in elderly adults: the potential mediating role of serum neurofilament light chain levels
Source: Front Nutr. 2025 Jan 29;12:1485648. doi: 10.3389/fnut.2025.1485648 (PMC11813793; doi:10.3389/fnut.2025.1485648)
Supplement: Supplementary file 1 [file Data_Sheet_1.docx]

**Supplementary Material**

**Individual and combined effects of dietary vitamin intakes on cognitive function in elderly adults: the potential mediating role of serum neurofilament light chain levels**

Zhikui Zhou ^a^, Baiyun Fan ^a^, Qiang Chen ^a^, Xuezhong Li ^a^, Xianjin Ke ^b*^

^a^ Department of Neurology, Affiliated People's Hospital of Jiangsu University, Zhenjiang, China.

^b^ Department of Neurology, Affiliated Hospital of Jiangsu University, Zhenjiang, China.

| **FIGURE S1** | Flowchart of study participants | 2 |
| --- | --- | --- |
| **FIGURE S2** | Directed acyclic graph showing the assumed causal relationship between vitamins and cognitive function | 3 |
| **FIGURE S3** | Dose-response relationships between individual vitamin intake and global cognitive function | 4-5 |
| **TABLE S1** | Coefficients and 95% confidence intervals for associations between specific cognitive function and single vitamin intake | 6 |
| **TABLE S2** | Associations between serum neurofilament light levels or global cognitive function and single vitamin intake | 7 |
| **TABLE S3** | Associations between serum neurofilament light levels or global cognitive function and single vitamin intake in source population | 8 |

**
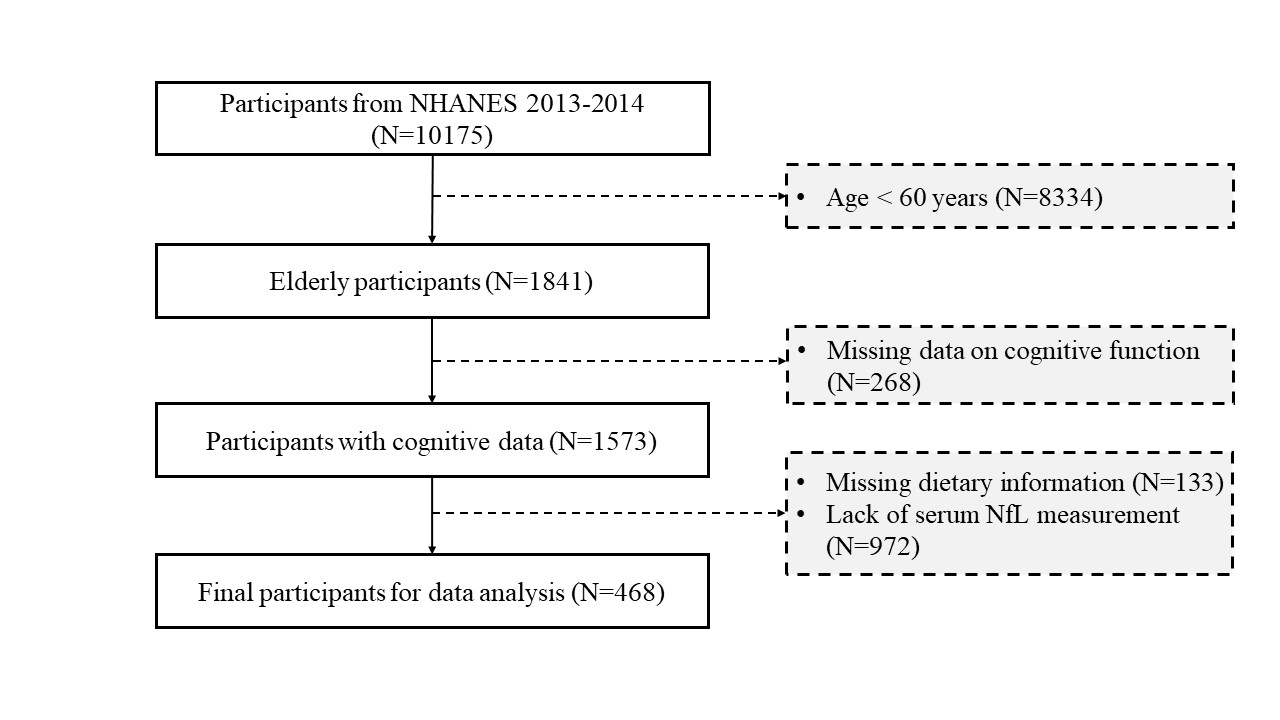
FIGURE S1:** Flowchart of study participants

**
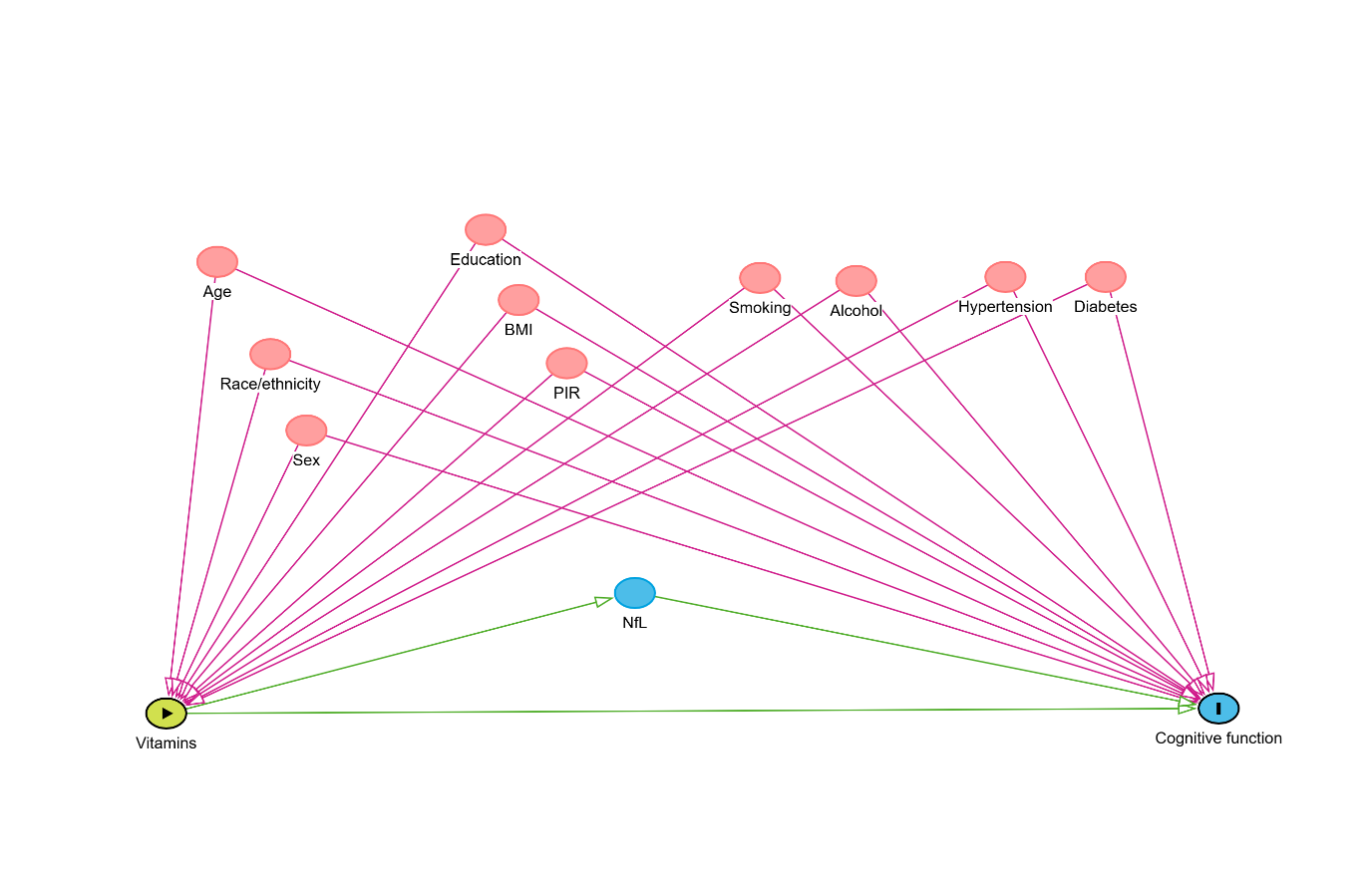
FIGURE S2:** Directed acyclic graph showing the assumed causal relationship between vitamins and cognitive function

**
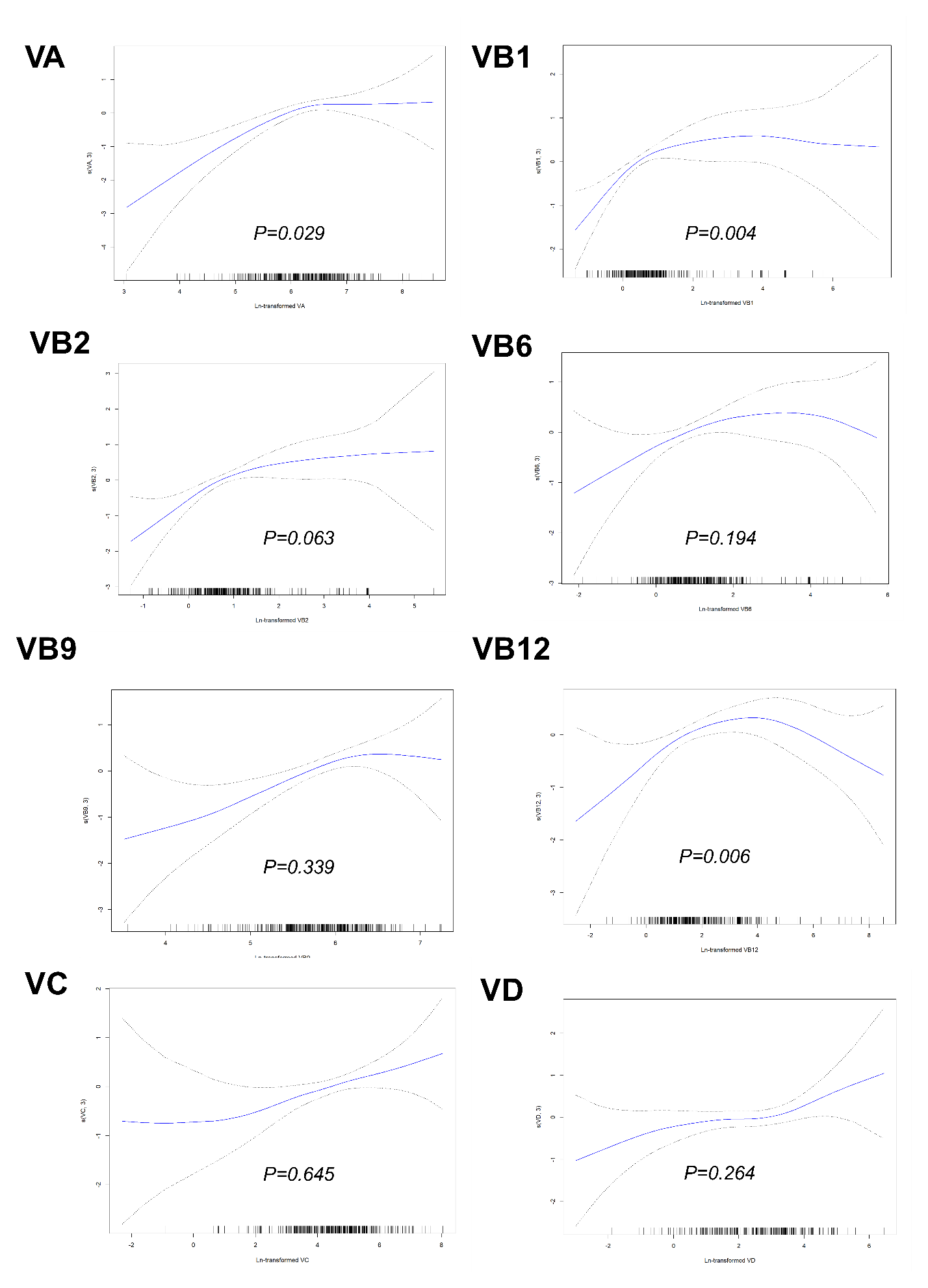
**

**
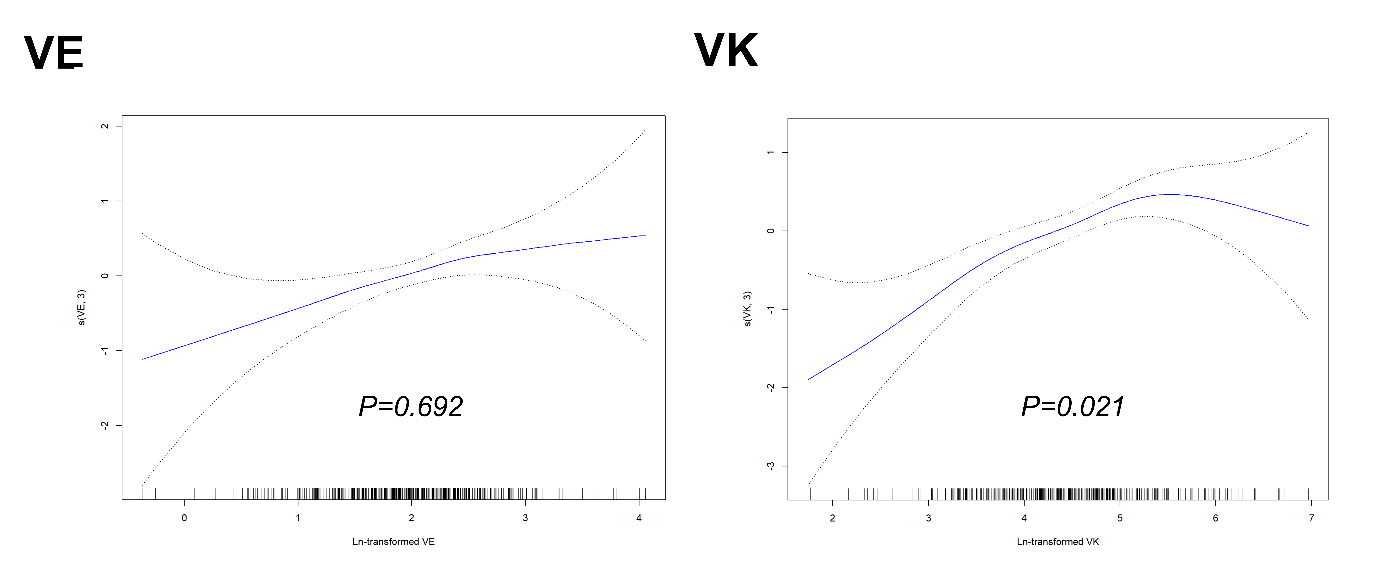
**

**FIGURE S3.** Dose-response relationships between individual vitamin intake and global cognitive function

**TABLE S1:** Coefficients and 95% confidence intervals for associations between specific cognitive function and single vitamin intake

|  | **CERAD Z score** | | |  | **AFT Z score** | | |  | **DSST Z score** | | |
| --- | --- | --- | --- | --- | --- | --- | --- | --- | --- | --- | --- |
|  | **Tertile 1** | **Tertile 2** | **Tertile 3** |  | **Tertile 1** | **Tertile 2** | **Tertile 3** |  | **Tertile 1** | **Tertile 2** | **Tertile 3** |
| Vitamin A | Ref. | 0.24 (0.03, 0.46) | 0.25 (0.02, 0.47) |  | Ref. | 0.36 (0.15, 0.57) | 0.07 (-0.15, 0.29) |  | Ref. | 0.18 (0.00, 0.34) | 0.17 (-0.01, 0.35) |
| Vitamin B1 | Ref. | 0.12 (-0.11, 0.34) | 0.03 (-0.21, 0.26) |  | Ref. | 0.28 (0.06, 0.50) | 0.19 (-0.03, 0.42) |  | Ref. | 0.18 (0.01, 0.36) | 0.14 (-0.04, 0.32) |
| Vitamin B2 | Ref. | 0.08 (-0.14, 0.30) | 0.10 (-0.14, 0.34) |  | Ref. | 0.19 (-0.03, 0.41) | 0.27 (0.04, 0.51) |  | Ref. | 0.11 (-0.07, 0.28) | 0.20 (0.01, 0.39) |
| Vitamin B6 | Ref. | 0.05 (-0.16, 0.27) | 0.05 (-0.17, 0.27) |  | Ref. | 0.06 (-0.15, 0.28) | 0.15 (-0.07, 0.37) |  | Ref. | 0.15 (-0.02, 0.33) | 0.16 (-0.02, 0.34) |
| Vitamin B9 | Ref. | 0.10 (-0.11, 0.33) | 0.13 (-0.13, 0.39) |  | Ref. | 0.10 (-0.12, 0.32) | 0.31 (0.05, 0.56) |  | Ref. | 0.23 (0.05, 0.40) | 0.24 (0.04, 0.45) |
| Vitamin B12 | Ref. | 0.05 (-0.16, 0.26) | 0.05 (-0.16, 0.27) |  | Ref. | 0.14 (-0.07, 0.35) | 0.14 (-0.07, 0.35) |  | Ref. | 0.12 (-0.05, 0.29) | 0.09 (-0.07, 0.26) |
| Vitamin C | Ref. | 0.03 (-0.17, 0.24) | 0.02 (-0.20, 0.24) |  | Ref. | 0.20 (0.00, 0.41) | 0.29 (0.07, 0.51) |  | Ref. | -0.03 (-0.20, 0.13) | 0.09 (-0.08, 0.27) |
| Vitamin D | Ref. | -0.02 (-0.23. 0.19) | -0.06 (-0.28, 0.16) |  | Ref. | 0..05 (-0.16, 0.26) | 0.12 (-0.09, 0.34) |  | Ref. | 0.08 (-0.08, 0.25) | 0.08 (-0.09, 0.25) |
| Vitamin E | Ref. | 0.01 (-0.21, 0.23) | 0.06 (-0.21, 0.33) |  | Ref. | 0.12 (-0.10, 0.33) | 0.25 (-0.01, 0.52) |  | Ref. | 0.02 (-0.16, 0.20) | 0.14 (-0.07, 0.35) |
| Vitamin K | Ref. | 0.16 (-0.05, 0.39) | 0.28 (0.05, 0.52) |  | Ref. | 0.11 (-0.09, 0.33) | 0.33 (0.09, 0.56) |  | Ref. | 0.14 (-0.03, 0.32) | 0.29 (0.11, 0.48) |

Models were adjusted by age, gender, race/ethnicity, education, family income to poverty ratio, body mass index, marital status, smoking status, alcohol consumption, physical activity, hypertension, diabetes, and energy intake

**TABLE S2:** Associations between serum neurofilament light levels or global cognitive function and single vitamin intake with adjusting for beta-carotene

|  | **Serum neurofilament light levels** | | | | |  | **Global cognitive function** | | | | |
| --- | --- | --- | --- | --- | --- | --- | --- | --- | --- | --- | --- |
|  | **Tertile 1** | **Tertile 2** | | **Tertile 3** | |  | **Tertile 1** | **Tertile 2** | | **Tertile 3** | |
|  |  | β (95%CI) | *p*-value | β (95%CI) | *p*-value |  |  | β (95%CI) | *p*-value | β (95%CI) | *p*-value |
| Vitamin A | Ref. | 0.03 (-0.10, 0.16) | 0.656 | 0.07 (-0.08, 0.23) | 0.372 |  | Ref. | 0.63 (0.17, 1.08) | 0.008 | 0.36 (-0.19, 0.91) | 0.196 |
| Vitamin B1 | Ref. | -0.02 (-0.14, 0.10) | 0.784 | -0.05 (-0.20, 0.09) | 0.463 |  | Ref. | 0.15 (-0.28, 0.58) | 0.496 | 0.43 (-0.08, 0.94) | 0.102 |
| Vitamin B2 | Ref. | -0.10 (-0.23, 0.02) | 0.105 | -0.02 (-0.16, 0.13) | 0.832 |  | Ref. | 0.39 (-0.04, 0.83) | 0.078 | 0.35 (-0.15, 0.85) | 0.174 |
| Vitamin B6 | Ref. | -0.02 (-0.15, 0.10) | 0.705 | -0.12 (-0.26, 0.03) | 0.121 |  | Ref. | 0.15 (-0.30, 0.60) | 0.516 | 0.39 (-0.13, 0.91) | 0.140 |
| Vitamin B9 | Ref. | -0.08 (-0.20, 0.05) | 0.243 | 0.01 (-0.13, 0.16) | 0.857 |  | Ref. | 0.31 (-0.14, 0.76) | 0.177 | 0.73 (0.23, 1.24) | 0.005 |
| Vitamin B12 | Ref. | 0.00 (-0.12, 0.13) | 0.969 | -0.07 (-0.20, 0.06) | 0.307 |  | Ref. | 0.13 (-0.31, 0.57) | 0.562 | 0.47 (0.01, 0.94) | 0.046 |
| Vitamin C | Ref. | 0.08 (-0.05, 0.20) | 0.225 | 0.07 (-0.06, 0.21) | 0.292 |  | Ref. | 0.28 (-0.17, 0.72) | 0.221 | 0.21 (-0.27, 0.69) | 0.400 |
| Vitamin D | Ref. | -0.01 (-0.13, 0.11) | 0.880 | 0.04 (-0.08, 0.17) | 0.510 |  | Ref. | 0.34 (-0.08, 0.77) | 0.111 | 0.07 (-0.36, 0.51) | 0.743 |
| Vitamin E | Ref. | -0.03 (-0.16, 0.10) | 0.656 | -0.10 (-0.25, 0.04) | 0.165 |  | Ref. | 0.05 (-0.40, 0.50) | 0.830 | 0.41 (-0.11, 0.93) | 0.125 |
| Vitamin K | Ref. | -0.17 (-0.30, -0.04) | 0.011 | -0.13 (-0.29, 0.03) | 0.102 |  | Ref. | 0.46 (-0.01, 0.93) | 0.057 | 0.76 (0.20, 1.32) | 0.008 |
| Models were adjusted by age, gender, race/ethnicity, education, family income to poverty ratio, body mass index, marital status, smoking status, alcohol consumption, physical activity, hypertension, diabetes, energy intake and beta-carotene | | | | | | | | | | | |

**TABLE S3:** Associations between serum neurofilament light levels or global cognitive function and single vitamin intake in source population

|  | **Serum neurofilament light levels (n = 736)** | | | | |  | **Global cognitive function (n = 1440)** | | | | |
| --- | --- | --- | --- | --- | --- | --- | --- | --- | --- | --- | --- |
|  | **Tertile 1** | **Tertile 2** | | **Tertile 3** | |  | **Tertile 1** | **Tertile 2** | | **Tertile 3** | |
|  |  | β (95%CI) | *p*-value | β (95%CI) | *p*-value |  |  | β (95%CI) | *p*-value | β (95%CI) | *p*-value |
| Vitamin A | Ref. | 0.03 (-0.07, 0.12) | 0.579 | 0.08 (-0.03, 0.18) | 0.158 |  | Ref. | 0.45 (0.20, 0.70) | <0.001 | 0.39 (0.13, 0.66) | 0.004 |
| Vitamin B1 | Ref. | -0.01 (-0.10, 0.08) | 0.802 | -0.05 (-0.17, 0.08) | 0.453 |  | Ref. | 0.23 (-0.01, 0.48) | 0.065 | 0.24 (-0.05, 0.52) | 0.107 |
| Vitamin B2 | Ref. | -0.04 (-0.13, 0.05) | 0.405 | -0.00 (-0.13, 0.12) | 0.959 |  | Ref. | 0.46 (0.21, 0.71) | <0.001 | 0.38 (0.09, 0.66) | 0.011 |
| Vitamin B6 | Ref. | -0.02 (-0.12, 0.08) | 0.649 | -0.08 (-0.20, 0.05) | 0.227 |  | Ref. | 0.20 (-0.05, 0.45) | 0.118 | 0.32 (0.04, 0.60) | 0.025 |
| Vitamin B9 | Ref. | -0.01 (-0.10, 0.08) | 0.865 | 0.03 (-0.07, 0.13) | 0.575 |  | Ref. | 0.23 (-0.02, 0.48) | 0.075 | 0.43 (0.15, 0.70) | 0.002 |
| Vitamin B12 | Ref. | -0.02 (-0.10, 0.07) | 0.675 | -0.03 (-0.16, 0.11) | 0.696 |  | Ref. | 0.34 (0.10, 0.59) | 0.006 | 0.43 (0.16, 0.69) | 0.001 |
| Vitamin C | Ref. | 0.04 (-0.06, 0.14) | 0.405 | 0.08 (-0.03, 0.19) | 0.174 |  | Ref. | 0.15 (-0.10, 0.41) | 0.237 | 0.10 (-0.15, 0.35) | 0.443 |
| Vitamin D | Ref. | -0.01 (-0.10, 0.09) | 0.914 | 0.04 (-0.07, 0.14) | 0.515 |  | Ref. | 0.27 (0.02, 0.51) | 0.032 | 0.22 (-0.04, 0.47) | 0.094 |
| Vitamin E | Ref. | 0.04 (-0.07, 0.14) | 0.515 | -0.10 (-0.23, 0.03) | 0.155 |  | Ref. | 0.25 (-0.00, 0.51) | 0.052 | 0.59 (0.31, 0.88) | <0.001 |
| Vitamin K | Ref. | -0.08 (-0.18, 0.03) | 0.160 | -0.10 (-0.22, 0.03) | 0.132 |  | Ref. | 0.32 (0.07, 0.57) | 0.013 | 0.76 (0.50, 1.02) | <0.001 |
| Models were adjusted by age, gender, race/ethnicity, education, family income to poverty ratio, body mass index, marital status, smoking status, alcohol consumption, physical activity, hypertension, diabetes, and energy intake | | | | | | | | | | | |

Code for BKMR

attach(new)

library(bkmr)

library(ggplot2)

Sex<-as.factor(Sex)

Age<-as.numeric(Age)

Race<-as.factor(Race)

Edu<-as.factor(Edu)

PIR<-as.numeric(PIR)

BMI<-as.numeric(BMI)

ALQ<-as.factor(ALQ)

PA<-as.factor(PA)

Diabetes<-as.factor(Diabetes)

HBP<-as.factor(HBP)

Energy<-as.numeric(Energy)

SumZ<-as.numeric(SumZ)

covar <- as.matrix(new[, c("Sex","Age","Race","Edu","PIR","BMI","ALQ", "PA", "Diabetes", "HBP","Energy")])

expos<-as.matrix(new[,c("VA","VB1","VB2","VB6","VB9","VB12","VC","VD","VE","VK")])

colnames(covar) <- paste0("x", 1:ncol(covar))

colnames(expos) <- paste0("z", 1:ncol(expos))

scale_expos <- scale(expos)

set.seed(10000)

fitkm <- kmbayes(SumZ, Z = scale_expos, X= covar, iter = 5000,

verbose = FALSE, varsel = TRUE)

ExtractPIPs(fitkm)

pred.resp.univar <- PredictorResponseUnivar(fit = fitkm)

levels(pred.resp.univar$variable)

levels(pred.resp.univar$variable) <- c("VA","VB1","VB2","VB6","VB9","VB12","VC","VD","VE","VK")

ggplot(pred.resp.univar, aes(z, est, ymin = est - 1.96*se,

ymax = est + 1.96*se)) +

geom_smooth(stat = "identity") +

facet_wrap(~variable, ncol = 4) +

xlab("Vitamins") +

ylab("global cognitive function") +geom_rug(sides = "b", position='jitter')

risks.overall <- OverallRiskSummaries(fit = fitkm, qs = seq(0.25, 0.75, by = 0.05), q.fixed = 0.5)

risks.overall

ggplot(risks.overall, aes(quantile, est, ymin = est - 1.96*sd,

ymax = est + 1.96*sd)) +

geom_hline(yintercept = 0, lty = 2, col = "brown") +

geom_pointrange()+xlab("quartile of vitamins") +

ylab("Estimated difference in global cognitive function")

risks.singvar <- SingVarRiskSummaries(

fit = fitkm, qs.diff = c(0.25, 0.75),

q.fixed = c(0.25, 0.50, 0.75))

subset(risks.singvar, variable %in% c("VA","VB1","VB2","VB6","VB9","VB12","VC","VD","VE","VK"))

singvarrisk2<- risks.singvar

class(singvarrisk2$variable)

levels(singvarrisk2$variable)
levels(singvarrisk2$variable) <- c("VA","VB1","VB2","VB6","VB9","VB12","VC","VD","VE","VK")

ggplot(risks.singvar, aes(singvarrisk2$variable, est, ymin = est - 1.96*sd, ymax = est + 1.96*sd,

col = q.fixed)) + geom_hline(aes(yintercept=0), linetype="dashed", color="red")+

geom_pointrange(position = position_dodge(width = 0.75)) + coord_flip()+ggtitle("")+ scale_x_discrete(name=" Estimated difference in global cognitive function ")+ scale_y_continuous(name="Vitamins")

risks.int <- SingVarIntSummaries(fit = fitkm,

qs.diff = c(0.25, 0.75), qs.fixed = c(0.25, 0.75))

risks.int

ggplot(risks.int, aes(variable, est, ymin = est - 1.96*sd, ymax = est + 1.96*sd)) +

geom_pointrange(position = position_dodge(width = 0.75)) +

geom_hline(yintercept = 0, lty = 2, col = "brown") +

coord_flip()
